# Digital twins and the future of precision mental health

**DOI:** 10.3389/fpsyt.2023.1082598

**Published:** 2023-03-13

**Authors:** Michael Spitzer, Itai Dattner, Sigal Zilcha-Mano

**Affiliations:** ^1^Department of Psychology, University of Haifa, Haifa, Israel; ^2^Department of Statistics, University of Haifa, Haifa, Israel

**Keywords:** precision mental health, digital twins, personalized medicine, psychotherapy, mental health interventions

## Abstract

Science faces challenges in developing much-needed precision mental health treatments to accurately identify and diagnose mental health problems and the optimal treatment for each individual. Digital twins (DTs) promise to revolutionize the field of mental health, as they are doing in other fields of science, including oncology and cardiology, where they have been successfully deployed. The use of DTs in mental health is yet to be explored. In this Perspective, we lay the conceptual foundations for mental health DTs (MHDT). An MHDT is a virtual representation of an individual’s mental states and processes. It is continually updated from data collected over the lifespan of the individual, and guides mental health professionals in diagnosing and treating patients based on mechanistic models and statistical and machine learning tools. The merits of MHDT are demonstrated through the example of the working alliance between the therapist and the patient, which is one of the most consistent mechanisms predicting treatment outcome.

## 1. Introduction

Science faces challenges in developing much-needed precision mental health treatments. In this Perspective, we discuss the potential of using digital twin (DT) technology for advancing clinical research and treatment of mental health. Although the concept of the DT has existed for more than a decade, there still is confusion about what it is (1). We begin with a brief description of DTs, then we discuss potential applications of the technology in mental health.

### 1.1. Digital twin

A DT is a virtual entity designed to represent, in as much detail as possible, a physical one. A virtual representation of this type makes possible better design and control of physical entities over their lifetime. The DT concept has received growing attention both in academia and industry (2–5), and a recent Gartner survey revealed that DTs are entering mainstream use (6). Although a variety of disciplines have adopted DTs, there is no generally accepted definition of DT, each discipline providing its own definition. For example, in agriculture, DT is considered “a dynamic virtual representation of a physical object or system, usually across multiple stages of its lifecycle, that uses real-world data, simulation, or machine learning models combined with data analysis to enable understanding, learning, and reasoning. DT can be used to answer what-if questions and should be able to present insights in an intuitive way” (7). The main differentiator of DTs from typical simulators or recommendation systems in agriculture (8) is that typical simulators are offline and recommendation systems are usually not based on physical models but only statistical/machine learning algorithms. By contrast, DTs span the lifecycle of an individual or process, are updated from real data, and use physical and mechanistic models, statistical/machine learning, and artificial intelligence (AI) to provide evidence-based guidance for the user. The above notions derived from industry and agriculture seem sufficiently general to fit other domains, such as mental health.

### 1.2. Potential benefits of DT in mental health?

DT equips its users with advanced decision-making capabilities using a continually updated virtual representation of reality. In mental health treatment, advanced capabilities may include the ability to *design* better treatments by simulating potential therapeutic scenarios *before* applying the treatment in reality, and providing online feedback and recommendations to the therapist for *optimizing* a treatment *within* a treatment period. DTs can produce what precision mental health needs: forecast deterioration in mental health and predict the process and outcome of mental health interventions. Based on this knowledge, DT can determine the most effective treatment for any given individual. It may sound like science fiction, but the technology is already in place. In mental health, DTs can produce patient-specific predictions for diagnosis, prognosis, treatment selection, and treatment tailoring.

DTs reproduce an individual’s functioning and behavior by generating real-time replicas of individual emotions, cognitions, and behaviors, updated in real time using data collected from various sources (e.g., sensors, questionnaires). This individual-specific knowledge can serve to monitor mental health status, determine clinical diagnosis, and issue alarms when intervention is needed. Statistical and physical mechanistic models make possible robust, interpretable, and reproducible analysis of mental health data and can infer missing states and parameters. A DT can serve to test various types of treatments that differ in their mechanisms of action and identify the one showing the best outcome for a given individual. Virtually testing different treatments has the advantage of cutting long, expensive trial-and-error processes, experimenting with various treatments until one is found that fits the individual’s characteristics and is effective. The DT can not only identify the most effective treatment for the individual, but also formulate recommendations on how to tailor the treatment to the patient based on data collected in real time.

In general, a DT applied in the mental health domain should provide the following functions in real time:

**Monitoring**: tracking the mental state of individuals and informing them about changes in it. With the patients’ informed consent, they can present the results to stakeholders (e.g., HMOs), issuing alarms when detecting a deterioration. DTs can signal the need for preventive intervention when deterioration is expected to persist, and predict the potential effect of a stress-evoking future event, such as a test for a student or deployment for a soldier.

**Diagnostics**: diagnosing mental health disorders and comorbidities, and tracking their development and fluctuations.

**Prognostics**: predicting the course of underlying processes by combining real and synthetic data with empirical and mechanistic models, using online simulations and root-cause analyses.

**Guidance**: indicating actions to take, for example, recommending the optimal treatment and the most effective techniques to use with a patient based on all the available options.

A useful DT should be a virtual entity reflecting in detail the mechanism and dynamic nature of the patient’s mental health and pathophysiology of disturbance in mental health, as well as the therapeutic processes and patient-therapist quality of relationship in the therapy room. A virtual representation can equip both patient and therapist with powerful tools enabling optimal treatment based on transparent reasoning and probabilistic considerations, generalizing insights learned in the past to new situations, not necessarily encountered before for a particular patient or therapist.

DTs have been successfully used in areas where validated physical models of the phenomenon of interest exists, such as industrial or agricultural applications. Mature and validated physical models together with data (e.g., from IoT sensors) and machine learning and AI algorithms have formed an ideal environment for the deployment of DTs. The question is whether DT technology is relevant for the mental health domain where at least some of the phenomena of interest are behavioral, and physical models are less common. In the next section we point out an example where a positive answer is plausible and lay the conceptual foundations for the promising approach of Mental Health DTs.

## 2. Mental health digital twins

Mental health is a state of mental wellbeing that enables people to cope with the stresses of life, realize their abilities, learn and work well, and contribute to their community (WHO). Mental health is determined by a complex interplay between individual, social, and structural stresses. The physical state-space representing the actual state of the mental health of an individual is a complex high-dimensional space that typically cannot be fully observed or directly measured and modeled. Thus, developing an exact or even approximate virtual replica of the entire mental health of an individual is an ambitious long-term research goal, if it is feasible at all.

Consider major depressive disorder (MDD), the leading cause of disability worldwide and a main contributor to the overall global burden of disease (9). Hundreds of active treatments are available for MDD, differing in their underlying mechanisms and how they are theorized to drive therapeutic change. But they do not appear to differ in their efficacy, which is around 50% for the “average patient” (10). Yet, some subpopulations of patients show great ability to benefit from a given treatment, whereas others are less able to do so (11). Despite advances in mental health interventions, there has been little change in overall treatment efficacy in the past five decades (10). Further focusing our mental health target to “twin,” we consider the therapeutic alliance between the patient and therapist in psychotherapy, which is one of the most consistent predictors of treatment outcomes in psychotherapy research (12, 13). We refer to the therapeutic alliance as the physical “assets” for which “twinning” may be possible.

### 2.1. Therapist-patient alliance digital twin

The therapist-patient alliance is the relationship that forms between therapist and patient during treatment, potentially a collaborative relationship built on mutual trust and understanding. Patient come to treatment with different capacities to realize this potential, which may change in the course of treatment and bring cure in itself. This alliance is considered to be a key factor in the success of the therapy because it allows the therapist to understand the patient’s needs and motivations, and patients to feel comfortable and safe in discussing and working through their challenges and goals. A common definition of alliance is the one proposed by Bordin (14), emphasizing three main aspects: agreement on the goals and tasks of therapy, a bond between therapist and patient, and a shared understanding of the therapeutic process. These aspects can work together to create a therapeutic environment conducive to change and growth.

Decades of empirical research suggest that a stronger alliance is significantly associated with better treatment outcomes (15). Recently (16), the importance of disentangling two distinct components of alliance has been demonstrated: the patients’ general tendency to form satisfying relationships with others, which affects also the relationship with the therapist (trait-like component of alliance), and the changes in such tendencies through interaction with the therapist (state-like component of alliance). The former enables treatment to be effective; the latter makes alliance therapeutic.

In view of the above scientific findings, developing an Alliance Digital Twin (ADT) seems to be a timely and useful technology to have at hand. The ultimate goal of ADT technology is to assist the therapist in improving the therapeutic alliance and consequently, treatment outcome. To demonstrate the great potential of ADT, we conceptualize a hypothetical example based on established and well-studied ingredients. Our goal is to demonstrate a concept; therefore, we made the example as simple as possible, but not simpler.

**Figure 1:**
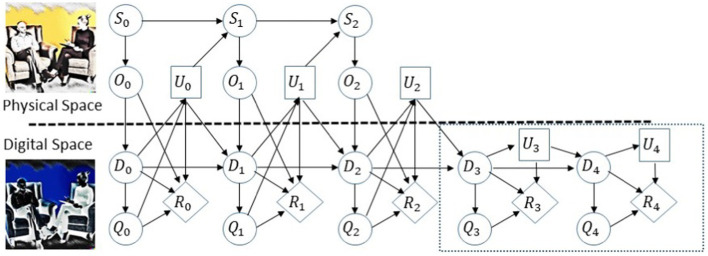
Probabilistic graphic model for the ADT, adapted from Kapteyn et al. (17). The upper path of the graphical model represents the time evolution of the physical asset states, denoted by S; the lower path represents the time evolution of the digital states, denoted by D. The graphical model encodes the coupling over time between an asset and its DT. At each time point, we measure the physical asset and this information flows into the DT denoted by O. The observational data are used to update the digital states D and the models comprising the DT, which, in turn, are used to predict quantities of interest Q. Then, based on model predictions a decision or control input U is chosen and applied to the physical asset. The above quantities and actions influence the reward denoted by R.

Following Kapteyn et al. (17), we view the physical asset (the alliance) and its DT as two coupled dynamical systems, evolving over time through their respective state spaces. Note that there are at least two time scales to consider: (a) session time (possibly weekly), and (b) time within a session, where resolution can be as fine as minutes or seconds. We use a probabilistic graphical model (dynamic Bayesian network with the addition of decision nodes) to define the elements comprising this coupled dynamical system over sessions of therapy, and the interactions that need to be modeled in the DT. Figure 1 shows a visual representation of the probabilistic graphical model for the asset-twin system.

The time index in Figure 1 corresponds to consecutive therapeutic sessions that take place over, say, consecutive weeks. The conditional independence structure defined by the graph allows us to factorize joint distributions over variables in the model (nodes in the graph can, in general, represent multivariate random variables) and for end-to-end uncertainty quantification and principled analysis, prediction, and decision-making. The methodology also allows predicting the dynamics ahead of time to make the optimal decision. For example, in Figure 1, in the dynamic network from time step 3 in the dashed rectangle, no observations about the physical states are made, but only forecasts in the digital space are generated, so an optimal decision can be made in time step 2.

The first stage in designing the ADT is to define the components of the graph depicted in Figure 1 and its structure by a topological reorganization of the graph, as will be required for the use case at hand. To this end, specific physical quantities to be measured must be defined. Natural candidates for that purpose can be motivated by existing psychotherapy research. We consider three quantities: (a) the efficacy and effectiveness of treatment, which in the case of depression can be measured by the Hamilton Rating Scale for Depression (HRSD) (18); (b) the quality of the therapeutic alliance in each session, measured by self-report questionnaires completed by the patient and the therapist, such as the Working Alliance Inventory (WAI; (19)); and (c) the presence and magnitude of ruptures in the alliance measured during treatment, for example, by nonverbal synchrony using quantification of motion energy (20). The theoretical and clinical literature, as well as empirical studies, suggests that one of the most promising processes underlying the effect of alliance on outcome are episodes of ruptures and repair in the alliance (21). Ruptures are defined as deterioration or tension in the alliance, manifested by a disagreement between the patient and therapist on treatment goals, lack of collaboration on therapeutic tasks, or strain on their emotional bond (21, 22). Ruptures are an integral part of treatment and take place in 91%–100% of therapy sessions. When resolved, ruptures are associated with better treatment outcomes; when not, they can become a risk for treatment failure, as manifested in patients’ deterioration and dropout. To resolve a rupture, therapists need to first identify it, then implement certain resolution strategies to repair it. Therapists often appear to miss ruptures occurring during treatment, relative to those found in observer-based coding (23), which further motivates the development of a technology allowing for real-time detection of ruptures. For example, a recent study (24) demonstrated that nonverbal synchrony, characterized by motion synchronization of therapists and patients during a session, can be used as a marker of alliance ruptures. The potential merits of focusing on patient-therapist synchrony in psychotherapy are discussed in the literature (25).

Summarizing the quantities of interest:

– Assessment of treatment efficacy and effectiveness measured by HRSD values of patients throughout the therapy at the beginning of each session (18).
– Strength of the alliance in each session, measured at the end of the session by WAI (19).
– Ruptures occurring during sessions, measured by nonverbal synchrony by quantification of motion energy (20) and tracked during each session with a resolution of minutes.

These quantities can be used to construct the ADT as follows. The physical states (S) of the physical asset we are “twinning” is the quality of the alliance. The observational data (O) we have are the HRSD, WAI, and nonverbal synchrony. The digital states (D) are the HRSD and WAI values predicted by a pre-trained statistical/machine learning model that predicts these quantities using current and forecasted values of nonverbal synchrony measured during sessions. Forecasting future nonverbal synchrony values can be done, for example, using mechanistic models of synchrony (26, 27) characterizing the mechanism and dynamics underlying the motion of therapist and patient during the session. Such physical models can be fitted in real time to motion data to simulate future scenarios of synchronization levels or going in and out synchronization, which, in turn, may indicate an evolving rupture, which if addressed in real time, can strengthen the alliance and result in better treatment outcome. Thus, the digital state consists of a mechanistic model, real-time estimates of its parameters that have a psychological meaning, and a pre-trained machine learning model. The learning model takes the values predicted by the mechanistic model and infers psychotherapy quantities of interest (Q) describing the assets, which are estimated by model outputs such as confrontational and withdrawal ruptures, the quality of the alliance, and the overall efficacy of treatment. The actions or decisions (U) stand for the intervention carried out by the therapist within or between sessions to influence the physical asset, namely, improving the alliance. Finally, the reward (R) quantifies the overall performance of the asset-twin system that can be achieved by measuring the improvement in HRSD from session to session.

The definition of the above components of the asset-twin system is the first stage in the design and development of the ADT. In particular, in defining the digital state, we must consider what information is sufficient to support the use case at hand. Typically, the digital state-space is only a subset of the complex high-dimensional physical state-space, consisting of (simple but not simpler) models capturing variations in the physical asset that are relevant for diagnosis, prediction, and decision-making in the application of interest (17). Scientific methodologies developed in recent years for statistical learning of dynamical systems and for analyzing big data and measurement error models (28–39) are highly relevant for developing the computational models and statistical learning algorithms comprising the ADT; see also the recent review and references therein (40).

The above DT conceptualization allows for dynamically updating the computational models and integrating them within the data-driven analysis and decision-making feedback loop. The ADT can be validated in lab and clinical experiments.

## 3. Additional considerations in building MHDTs

Developing a DT requires following best statistical practices for data collection (observational and controlled experiments) and management (cleansing, validation, handling missing data, preprocessing). Another key element is the development of AI techniques that integrate mechanistic models describing the dynamics of mental health and therapeutic processes of patients going through treatment with advanced statistical/machine learning methods applied to various data sources such as motion, sound, social media activity, self-reports, etc. Such models can maximize interpretability, generalizability, and robustness by integrating data-driven results with conceptual models of psychopathology and psychotherapy [e.g., (41)], and accumulating empirical knowledge. They enable what-if analysis in real time by generating probable future paths of the underlying mental health dynamics. This, in turn, allows choosing the optimal intervention at a given time for individual patients. Our increasing capacity to analyze, integrate, and exploit many sources of high-dimensional data has firmly established our ability to use innovative data science techniques in constructing an individual’s DTs. The potential of implementing innovative data science advances to the field of mental health has been firmly established in recent years (42).

Below we describe the steps needed to develop, validate, and maintain ADT to characterize both individuals (patient, therapist) and therapeutic processes. We describe typical steps required when developing advanced technology based on domain knowledge, data, and statistical/machine learning algorithms.

**Step 1. Data collection**. The development of DT technology requires vast heterogeneous multidomain and multiscale spatiotemporal mental health data. Both data collected automatically and reported by individuals using ecological momentary assessments (EMA) methods can be included. Examples of automatically collected data include physiological measures gathered moment by moment using wearable technology, social media activity, sleep and motion patterns, acoustic vocal indices, etc. Data are handled remotely using cloud storage to enable real-time processing, and stored using HIPAA-compliant methods. Observational data and controlled experiment data should be approached differently. Indeed, for better repeatability, reproducibility, and generalization of research results, the data generation process of each data source should be well understood and rigorous protocols for data collection should be developed. Sources of statistical measurement error, bias, and variance should be studied and controlled if possible. Some examples are between and within (over time) bias and variance in sensors, individual, domain, and environment conditions. In particular, when learning dynamical systems is of interest, appropriate methodologies for design of experiments should be used given the underlying dynamic processes and the different time scales involved as discussed above.

**Step 2. Data management.** The development of DTs requires design and implementation of data pipelines in which the data flow from measurement (sensors, other) of the physical entity, to the virtual representation in the DT brain, resulting in alerts and recommendations for real-time or future interventions, spanning the life cycle of the physical entity or processes. Such data pipelines should be robust to be safely deployed in production environments, but flexible enough to adapt to new process, data, and algorithm requirements. In both research and production environments, the data pipelines should perform data cleansing, validation, and preprocessing. Ideally, such pipelines and processes should be fully automated, especially in production environments to allow for scaling. Specifically, the research stage usually starts with data preparation for analysis and exploratory data analysis (EDA). Data preparation typically includes data cleansing, validation, and preprocessing (normalization, transformations, baseline correction, manual and automatic outlier detection, handling missing data, etc.). In EDA, a variety of methods can be used, such as model-free data mining (e.g., association rules (43)), unsupervised methods (e.g., clustering (44)), fuzzy sets, rule-based reasoning, as well as the development of statistical and mathematical models characterizing the data generation process at all spatiotemporal scales. This step results in data pipelines and processes enabling advanced analysis and the development of physical/mechanistic models, statistical/machine learning, and AI models and algorithms.

**Step 3. Modeling.** Powerful DTs are based on modeling (e.g., mathematical, statistical, and symbolic) the dynamics of the physical entity within its environment. Such models have recently been developed by integrating statistical/machine learning and AI algorithms with mechanistic models describing the dynamics of the underlying process of interest. These models allow more interpretable inferences and predictions when applied to the data streamed into the DT. They also allow performing what-if analysis in real time by generating probable (in a well-defined probabilistic sense) future paths for the underlying dynamics. This, in turn, allows choosing the optimal intervention at a given time for a particular individual. For instance, a model may focus on predicting mental health diagnosis or specific clusters of symptoms both at the between-individuals and the within-individual levels (e.g., labile affect, maladaptive interpersonal interactions, adverse behaviors, etc.). Furthemore, application of transfer learning and domain adaptation tools is crucial for generalizing lab results to the field.

## 4. Summary

The modeling techniques mentioned above are natural advances to the existing state-of-the-art supervised and unsupervised machine learning approaches that have recently been used to identify mental health markers. Indeed, the purpose of supervised learning is to develop a model where both the mental health diagnosis (e.g., MDD) and symptoms (covariates) are given, so that the model can make a prediction of the diagnosis in the future, when only the features are given. For example, supervised machine learning identifies pre-specified features that are associated with MDD symptom severity. Unsupervised learning uses statistical algorithms to uncover structures in the data, such as similarities and dissimilarities between individuals sharing the same diagnosis. One of the most promising examples of such an approach is the identification of distinct biotypes of a given mental health disorder, like MDD, by parsing the heterogeneity contained in markers of MDD, enabling detection of biologically derived subgroups with unique psycho-socio-biological profiles. Both machine learning approaches have the potential to contribute to the understanding of MDD pathophysiology, they have complementary limitations: supervised methods are limited by the accuracy of the prior knowledge they rely on, and unsupervised methods by the potential for unclear or uninterpretable derivation of data-driven subtypes, which reduces their likelihood of being implemented in clinical practice. Given these complementary limitations, combining the two with mechanistic models into a hybrid model (45) can maximize their ability to yield meaningful new knowledge on mental health biotypes. Hence, to maximize interpretability and generalizability, the data-driven results are integrated with commonly used conceptual models of psychopathology and psychotherapy. Current ML approaches implemented in the field of mental health focus on classifying and predicting markers of mental health, but their solutions remain uninterpretable. ADT would retain prediction accuracy and at the same time endow these models with explanatory features. Validation of such a technology can be carried out in and out of the lab, to be tested with actual people experiencing stress.

Finally, as with any new technology, important details remain to be resolved, and challenges to be addressed (46), including ethical considerations of patient safety and privacy (47); methodological and technical challenges in the deployment and maintenance of the DTs in and out of the lab; successful adaptation to new situations not encountered before; and continuous learning and improvement of performance. Digital twins in mental health can serve as a vital link between basic science and real-world applications, providing personalized and efficient solutions that foster wellbeing, taking advantage of the digital revolution.

## Author contributions

All authors listed have made a substantial, direct, and intellectual contribution to the work and approved it for publication.

## Funding

The writing of this manuscript was supported by the Israel Science Foundation (ISF, Grant no. 395/19 to SZ-M and Grant no. 1755/22 to I. Dattner). The funder has no role in the decision to publish, or preparation of the manuscript.

## Conflict of interest

The authors declare that the research was conducted in the absence of any commercial or financial relationships that could be construed as a potential conflict of interest.

## Publisher’s note

All claims expressed in this article are solely those of the authors and do not necessarily represent those of their affiliated organizations, or those of the publisher, the editors and the reviewers. Any product that may be evaluated in this article, or claim that may be made by its manufacturer, is not guaranteed or endorsed by the publisher.

## References

[1] Grieves M. Intelligent digital twins and the development and management of complex systems. Digital Twin. (2022) 2:8. doi: 10.12688/digitaltwin.17574.1

[2] Kenett RS Bortman J. The digital twin in industry 4.0: a wide-angle perspective. Qual Reliab Eng Int. (2022) 38:1357–66. doi: 10.1002/qre.2948

[3] Grieves M. Digital twin: manufacturing excellence through virtual factory replication. Digital Twin White Paper. (2014) 1:1–7.

[4] Niederer SA Sacks MS Girolami M Willcox K. Scaling digital twins from the artisanal to the industrial. Nat Comput Sci. (2021) 1:313–20. doi: 10.1038/s43588-021-00072-5 38217216

[5] Chinesta F Cueto E Abisset-Chavanne E Duval JL Khaldi FE. Virtual, digital and hybrid twins: a new paradigm in data-based engineering and engineered data. Arch Comput Methods Eng. (2020) 27:105–34. doi: 10.1007/s11831-018-9301-4

[6] Gartner Survey Reveals (2019). Digital twins are entering mainstream. Available at: https://www.gartner.com/en/newsroom/press-releases/2019-02-20-gartner-survey-reveals-digital-twins-are-entering-mai (Accessed June 8, 2022).

[7] Janssen SJ Porter CH Moore AD Athanasiadis IN Foster I Jones JW. Towards a new generation of agricultural system data, models and knowledge products: information and communication technology. Agric Syst. (2017) 155:200–12. doi: 10.1016/j.agsy.2016.09.017, PMID: 28701813 PMC5485661

[8] Zhai Z Martínez JF Beltran V Martínez NL. Decision support Systems for Agriculture 4.0: survey and challenges. Comput Electron Agric. (2020) 170:105256. doi: 10.1016/j.compag.2020.105256

[9] Friedrich MJ. Depression is the leading cause of disability around the world. JAMA. (2017) 317:1517. doi: 10.1037/0033-3204.43.3.292 28418490

[10] Cuijpers P. Four decades of outcome research on psychotherapies for adult depression: an overview of a series of meta-analyses. Can Psychol. (2017) 58:7–19. doi: 10.1037/cap0000096

[11] Zilcha-Mano S. Major developments in methods addressing for whom psychotherapy may work and why. Psychother Res. (2019) 29:693–708. doi: 10.1080/10503307.2018.1429691, PMID: 29409394

[12] Zilcha-Mano S. Is the alliance really therapeutic? Revisiting this question in light of recent methodological advances. Am Psychol. (2017) 72:311–25. doi: 10.1037/a0040435, PMID: 28481579

[13] Hatcher RL Barends AW. How a return to theory could help alliance research. Psychother Theory Res Pract Train. (2006) 43:292–9. doi: 10.1037/0033-3204.43.3.292, PMID: 22122100

[14] Bordin ES. Theory and research on the therapeutic working alliance: new directions In: Horvath AO Greenberg LS, editors. The working alliance: theory, research, and practice. Hoboken, NJ: Wiley (1994). 13–37.

[15] Flückiger C Wampold BE Horvath AO. The alliance in adult psychotherapy: a meta- analytic synthesis. Psychotherapy. (2018) 55:316–40. doi: 10.1037/pst0000172, PMID: 29792475

[16] Zilcha-Mano S. Is the alliance really therapeutic? Revisiting this question in light of recent methodological advances. Am Psychol. (2017) 72:311–25. doi: 10.1037/a0040435, PMID: 28481579

[17] Kapteyn MG Pretorius JV Willcox KE. A probabilistic graphical model Foundation for Enabling Predictive Digital Twins at scale. Nat Comput Sci. (2021) 1:337–47. doi: 10.1038/s43588-021-00069-0 38217207

[18] Hamilton M. Development of a rating scale for primary depressive illness. Br J Soc Clin Psychol. (1967) 6:278–96. doi: 10.1111/j.2044-8260.1967.tb00530.x 6080235

[19] Horvath AO Greenberg LS. Development and validation of the working Alliance inventory. J Couns Psychol. (1989) 36:223–33. doi: 10.1037/0022-0167.36.2.223

[20] Ramseyer FT. Motion energy analysis (MEA): a primer on the assessment of motion from video. J Couns Psychol. (2020b) 67:536–49. doi: 10.1037/cou0000407, PMID: 32614233

[21] Safran JD Muran JC. Negotiating the therapeutic alliance: a relational treatment guide. New York, NY: Guilford Press (2000).

[22] Eubanks CF Lubitz J Muran JC Safran JD. Rupture resolution rating system (3RS): development and validation. Psychother Res. (2019) 29:306–19. doi: 10.1080/10503307.2018.1552034, PMID: 30526383 PMC6408286

[23] Eubanks CF Sinai M Israel B Muran JC Safran JD. Alliance rupture repair: a meta- analysis. Psychotherapy. (2018) 55:508–19. doi: 10.1037/pst0000185, PMID: 30335462

[24] Deres-Cohen K Dolev-Amit T Peysachov G Ramseyer FT Zilcha-Mano S. Nonverbal synchrony as a marker of alliance ruptures. Psychotherapy. (2021) 58:499–509. doi: 10.1037/pst0000384, PMID: 34881925

[25] Koole SL Tschacher W. Synchrony in psychotherapy: a review and an integrative framework for the therapeutic alliance. Front Psychol. (2016) 7:862. doi: 10.3389/fpsyg.2016.00862 27378968 PMC4907088

[26] Noy L Dekel E Alon U. The mirror game as a paradigm for studying the dynamics of two. Proc Natl Acad Sci U S A. (2011) 108:20947–52. doi: 10.1073/pnas.1108155108 22160696 PMC3248496

[27] Dahan A Noy L Hart Y Mayo A Alon U. Exit from synchrony in joint improvised motion. PLoS One. 11:e0160747. doi: 10.1371/journal.pone.0160747 PMC5053605 27711185

[28] Dattner I Klaassen CAJ. Optimal rate of direct estimators in systems of ordinary differential equations linear in functions of the parameters. Electron J Stat. (2015) 9:1939–73. doi: 10.1214/15-EJS1053

[29] Dattner I. A model-based initial guess for estimating parameters in systems of ordinary differential equations. Biometrics. (2015) 71:1176–84. doi: 10.1111/biom.12348 26172865

[30] Vujacic I Dattner I Gonzalez J Wit E. Time-course window estimator for ordinary differential equations linear in the parameters. Stat Comput. (2015) 25:1057–70. doi: 10.1007/s11222-014-9486-9

[31] Dattner I Miller E Petrenko M Kadouri DE Jurkevitch E Huppert A. Modelling and parameter inference of predator–prey dynamics in heterogeneous environments using the direct integral approach. J R Soc Interface. (2017) 14:20160525. doi: 10.1098/rsif.2016.0525, PMID: 28053112 PMC5310726

[32] Vujacic I Dattner I. Consistency of direct integral estimator for partially observed systems of ordinary differential equations. Stat Probab Lett. (2018) 132:40–5. doi: 10.1016/j.spl.2017.08.013

[33] Dattner I Gugushvili S. Application of one-step method to parameter estimation in ODE models. Statistica Neerlandica. (2018) 72:126–56. doi: 10.1111/stan.12124 29937593 PMC5993282

[34] Yaari R Dattner I. simode: R package for statistical inference of ordinary differential equations using separable integral-matching. J Open Source Softw. (2019) 4:1850. doi: 10.21105/joss.01850

[35] Dattner I Ship H Voit EO. Separable nonlinear least-squares parameter estimation for complex dynamic systems. Complexity. (2020) 2020:11–2020. doi: 10.1155/2020/6403641 PMC8188859 34113070

[36] Dattner I Goldberg Y Katriel G Yaari R Gal N Miron Y. The role of children in the spread of COVID-19: using household data from Bnei Brak, Israel, to estimate the relative susceptibility and infectivity of children. PLoS Comput Biol. (2021) 17:e1008559. doi: 10.1371/journal.pcbi.1008559, PMID: 33571188 PMC7877572

[37] Dattner I Goldenshluger A Juditsky A. On deconvolution of distribution functions. Ann Statist. (2011) 39:2477–501. doi: 10.1214/11-AOS907

[38] Dattner I Reiser B. Estimation of distribution functions in measurement error models. J Stat Plan Inference. (2013) 143:479–93. doi: 10.1016/j.jspi.2012.09.004

[39] Dattner I Reiß M Trabs M. Adaptive quantile estimation in deconvolution with unknown error distribution. Bernoulli. (2016) 22:143–92. doi: 10.3150/14-BEJ626

[40] Dattner I. Differential equations in data analysis. WIREs Comput Stat. (2021) 13:e1534. doi: 10.1002/wics.1534

[41] Tschacher W Haken H. The process of psychotherapy. Berlin, Germany: Springer International Publishing (2019).

[42] Cohen ZD Delgadillo J DeRubeis RJ. Personalized treatment approaches In: Barkham M Lutz W Castonguay LG, editors. Bergin and Garfield’s handbook of psychotherapy and behavior change. 7th ed. Hoboken, NJ: Wiley Blackwel (2021)

[43] Shabtay L Fournier-Viger P Yaari R Dattner I. A guided FP-Growth algorithm for mining multitude-targeted item-sets and class association rules in imbalanced data. Inf Sci. (2021) 553:353–75. doi: 10.1016/j.ins.2020.10.020

[44] Yaari R Huppert A Dattner I. Data-driven clustering of infectious disease incidence into age groups. Stat Methods Med Res. (2022) 31:2486–99. doi: 10.1177/09622802221129041 36217843

[45] Champaney V Chinesta F Cueto E. Engineering empowered by physics-based and data-driven hybrid models: a methodological overview. Int J Mater Form. (2022) 15:1–14. doi: 10.1007/s12289-022-01678-4

[46] Rasheed A San O Kvamsdal T. Digital twin: values, challenges and enablers from a modeling perspective. IEEE Access. (2020) 8:21980–2012. doi: 10.1109/ACCESS.2020.2970143

[47] Bruynseels K Santoni de Sio F Van den Hoven J. Digital twins in health care: ethical implications of an emerging engineering paradigm. Front Genet. (2018) 9:31. doi: 10.3389/fgene.2018.00031 29487613 PMC5816748

